# Repetitive transcranial magnetic stimulation (rTMS) for catatonia– a case report

**DOI:** 10.1192/j.eurpsy.2022.1911

**Published:** 2022-09-01

**Authors:** C. Licht, S. Fuchs, A. Ruttmann, K. Richter, T. Hillemacher

**Affiliations:** Paracelsus Medical University Clinic Nuremberg, Psychiatry And Psychotherapy, Nuernberg, Germany

**Keywords:** Catatonia, repetitive transcranial magnetic stimulation, Supplementory Motor Area, schizophrénia

## Abstract

**Introduction:**

Catatonia is one of the most common severe motor syndromes, with an estimated prevalence among psychiatric inpatients of about 15 %. Benzodiazepines and electroconvulsive therapy (ECT) are the most widely studied treatment methods recommended as first-line therapy. We present the case of a 55-year-old female patient with paranoid schizophrenia and severe life-threatening catatonia who remitted under a short series of rTMS.

**Objectives:**

s. Introduction

**Methods:**

The point of resting motor threshold (RMT) for the musculus rectus femoris was determined for the left hemisphere. A straight line 3 cm anterior and parasagittal from that point defined the SMA. A total of three sessions, each with 1000 pulses at intensity 66 % of the RMT, were performed within 24 and 120 hours apart. Stimulation protocol was set to 1Hz in the area of the left SMA with 25 series of 40 pulses, pulse width 25 ms, angle of attack 45°. Hardware: MagVenture, 8-coil “cool-B65 butterfly-shaped coil from Medtronic.

**Results:**

Within 24 hours after the first session, a marked improvement of catatonic symptoms like independent locomotion and verbal communication was recognized. One week after the whole rTMS treatment, a food intake without a gastric tube was possible.

**Conclusions:**

The present case demonstrates that pronounced catatonia may be successfully treated with inhibitory rTMS. Our results underline the importance of non-invasive brain stimulation as a valuable addition to the existing treatment spectrum for catatonia. Future research is empowered to path the way for a significant expansion of treatment.

**Disclosure:**

No significant relationships.

